# Single-center analysis of servo-controlled cooling during the transport of neonates with perinatal asphyxia

**DOI:** 10.3389/fped.2025.1562736

**Published:** 2025-03-20

**Authors:** Lingzhu Huang, Qiru Su, Weimin Huang, Xueling Lu, Yu Lan Chen, Xue Yang, Jingbo Jiang

**Affiliations:** ^1^ College of Medicine, Shantou University, Shantou, Guangdong, China; ^2^ Clinical Research Department, Shenzhen Children's Hospital, Shenzhen, China; ^3^ Department of Neonatology, Shenzhen Children's Hospital, Shenzhen, China; ^4^ Department of Hyperbaric Oxygen Therapy, Shenzhen Children's Hospital, Shenzhen, China

**Keywords:** asphyxia, hypoxic-ischemic encephalopathy, therapeutic hypothermia, transport, servo-controlled cooling

## Abstract

**Objective:**

To investigate the safety and efficacy of servo-controlled cooling during the transport of neonates with perinatal asphyxia.

**Methods:**

We conducted a retrospective non-randomized case-control study at a single-center,which included 65 neonates diagnosed with Hypoxic-Ischemic Encephalopathy (HIE). These neonates were referred by the Shenzhen Children's Hospital medical transport team between January 2020 and June 2024. All subjects received 72 h of mild hypothermia treatment upon admission. Participants were categorized into an active group and a control group based on the use of servo-controlled cooling during transport. To evaluate differences in clinical characteristics, transport variables, and hospitalization outcomes between the two groups, we employed independent samples *t*-tests, Mann–Whitney *U* tests, and *χ*^2^ tests for inter-group comparison.

**Results:**

Among the 65 subjects, there were 42 males and 23 females. The active group comprised 17 patients, while the control group included 48. No statistically significant differences were observed in sex, gestational age, birth weight, or HIE grade between the two groups (*P* > 0.05). In comparison to the control group, the active group experienced a shorter duration from leaving the referral center to reaching the target temperature (1 h vs. 2.67 h, Z = −4.513, *P* < 0.05), arrived at the treatment center at a lower temperature (34.03°C vs. 35.6°C, t = −4.991, *P* < 0.05), and demonstrated a higher proportion of patients within the target temperature range upon arrival [88.2% (15/17) vs. 16.7% (8/48), *χ*^2^ = −0.774, *P* < 0.05]. Additionally, the length of hospitalization was shorter for the active group (15 days vs. 19 days, Z = −2.835, *P* < 0.05). The proportion of patients in the severe range on the aEEG recorded on the third day of cooling was higher in the control group [45.8% (22/48) vs. 11.8% (2/17), Z = −2.042, *P* < 0.05].

**Conclusion:**

Active therapeutic hypothermia during transport is both safe and feasible.It enables a more rapid and stable achievement of the target temperature, enhances short-term EEG outcomes, and may serve as the preferred method for transporting neonates with hypoxic-ischemic encephalopathy(HIE).

## Introduction

Hypoxic-Ischemic Encephalopathy (HIE) is a significant contributor to neonatal mortality and disability. Statistics indicate that the incidence of HIE is 0.15% in developed countries, while it ranges from 0.23% to 2.65% in developing countries. Approximately 20% of infants with HIE may die during the neonatalperiod, and 40% of survivors may experience neurological and other disabilities (1). Without treatment, 62% of neonates with HIE infants will develop moderate to severe disabilities or die by 18–22 months; however, this risk can be reduced to 41% with appropriate intervention (2, 3). Evidence suggests that controlled hypothermia therapy effectively reduces the likelihood of death or severe neurodevelopmental impairment in neonates with moderate to severe HIE, establishing it as the standard treatment for neuroprotection in this condition, with early initiation being critical (1, 4–7). However, managing moderate to severe HIE and implementing hypothermia requires multidisciplinary collaboration and advanced care (5). Some eligible HIE infants are born in centers lacking hypothermia treatment, which poses the risk of delays due to long-distance transfers that may exceed the recommended initiation timeline. Currently, there is limited research in China regarding the implementation and outcomes of hypothermia during transport for cases of asphyxia. This study analyzes the clinical data of asphyxia cases referred to our center, exploring the safety and efficacy of active hypothermia during transport, thereby providing a reference for clinical implementation.

## Data and methods

### Data collection

This retrospective non-randomized case-control study collected data from neonates with asphyxia who received controlled hypothermia treatment in the Neonatal Intensive Care Unit (NICU) of Shenzhen Children's Hospital from January 2020 to June 2024. The inclusion criteria were as follows (8, 9): (1) gestational age ≥35 weeks or birth weight ≥2,000 g; (2) evidence of hypoxia-ischemia meeting at least one of the following conditions: clear evidence of fetal distress (e.g., uterine rupture, placental abruption, abnormal fetal heart rate); 5 min Apgar score ≤5; resuscitation requiring positive pressure ventilation for more than 10 min; umbilical blood gas analysis indicating pH ≤7.10 or base excess ≤−12 mmol/L within one hour after birth; and (3) neurological assessment indicating moderate to severe HIE according to the modified Sarnat criteria (10). If mild HIE is diagnosed, the decision to initiate cooling should be made after consultation with a neonatal specialist. Exclusion criteria included: (1) severe congenital malformations; (2) traumatic brain injury or moderate to severe intracranial hemorrhage; (3) congenital metabolic diseases; (4) age at cooling initiation greater than 24 h or cessation of cooling within 72 h. Patients were categorized into two groups based on the use of servo-controlled cooling during transport: the Active Group, in which cooling was administered using a servo-controlled cooling device (Tecotherm Neo, Inspiration Healthcare, LTD; implemented at our center since September 2022), and the Control Group, in which infants were transported in incubators with temperatures maintained at 32–34°C without active cooling. The study received approval from the ethics committee of Shenzhen Children's Hospital (202403202), and informed consent was obtained from the guardians.

### Methods

Clinical Data: Information was gathered through a review of the hospital's electronic medical record system, focusing on three main categoies: (1) Baseline Data: gestational age, gender, birth weight, mode of delivery, Apgar score, and the first blood gas analysis (umbilical/arterial/venous) as well as the severity classification of HIE (10); (2) Transport Data: the age at which the neonate commenced hypothermia, th age at which the target temperature was achieved, the age upon arrival at the treatment center, the duration from departure at the referral center to achieving the target temperature (33°C-34°C), the temperature upon arrival at the treatment center, the proportion of neonates whose temperature fell within the target range upon arrival, heart rate, mean blood pressure, and blood gas analysis (pH, base excess) before and after transport; (3) Hospitalization Data: administration of vasopressors, duration of mechanical ventilation, length of hospital stay, seizures confirmed by electroencephalogram, amplitude-integrated electroencephalogram (aEEG) on days 1 and 3 of cooling, classification of HIE-related brain injury and affected areas on cranial MRI, and mortality rate.

All neonates underwent continuous video electroencephalogram (EEG) monitoring for four hours on the first and third days of cooling. This monitoring was performed at the bedside using the Nicolet Monitor (Natus, Middleton, WI, USA) and was conducted by trained neonatal specialist nurses. In accordance with the “Chinese Expert Consensus on the Clinical Application of aEEG in Neonates” (11), the aEEG was classified based on the upper and lower voltage limits of the background as follows: (1) Normal: upper boundary of the EEG activity amplitude spectrum ≥10 μV, lower boundary ≥5 μV; (2) Mildly abnormal: upper boundary >10 μV, lower boundary <5 μV; or normal amplitude with convulsive episodes; 3. Severely abnormal: upper boundary <10 μV, lower boundary <5 μV; or abnormal amplitude with convulsive episodes.

All neonates underwent cranial MRI examinations within 7–14 days after birth. These examinations included T1 and T2-weighted imaging, as well as diffusion-weighted series. In term neonates, two primary injury patterns associated with HIE are observable on MRI (12): (1) basal ganglia-thalamic pattern, which primarily affects the bilateral central gray nuclei (ventrolateral thalamus and posterior putamen) and the periventricular cortex; and (2) the watershed pattern, which involves the watershed areas (anterior-middle cerebral artery and posterior-middle cerebral artery), affecting the white matter. Based on the regions of brain injury, classifications include (12, 13): (1) deep gray matter (thalamus, basal ganglia, posterior limb of the internal capsule, brainstem, periventricular cortex, and hippocampus); (2) cerebral white matter/cortex (cortex, white matter, optic radiation, corpus callosum, punctate white matter lesions, and intraparenchymal hemorrhage); and (3) cerebellum (cerebellum and cerebellar hemorrhage).

### Implementation of hypothermia

In the Active Group, the transport team initiated active cooling immediately upon arrival at the referral center. Cooling was maintained continuously during transport, alongside intensive care management that included respiratory support, hemodynamic stabilization, and seizure control. A servo-controlled cooling device, which could be securely mounted on the transport platform, was utilized. The neonate was positioned on the mattress of the transport incubator with minimal clothing. The target temperature was achieved by circulating water through the device and the mattress. A core temperature monitoring probe was connected to one end of the device and inserted 4–5 cm into the infant's rectum at the other end. The device effectively maintained the set temperature range of 33℃–34℃ by continuously monitoring the infant's rectal temperature (14). Throughout the process, the nursing staff ensured that the temperature probe remained securely in place.

In cases where the asphyxiated neonate initially did not meet the established criteria for cooling, the neonate's clinical condition was closely monitored throughout transport. The evolution of the condition was dynamically assessed to determine the necessity for cooling intervention. Due to the early manifestation of symptoms and the limited diagnostic capabilities at the referral center, the severity of HIE could not be promptly evaluated. If there was significant suspicion of potential progression to moderate or severe HIE, cooling intervention was initiated following consultation with a neonatal specialist. Upon arrival at the NICU, aEEG was employed to assist in assessing the severity of HIE (15).

In the control group, neonates were transported in an incubator set to 32℃–34°C, receiving only intensive care management without any controlled cooling until their arrival at the NICU, where hypothermia treatment was subsequently initiated.

During transport, respiratory support was customized according to the neonate's respiratory condition, utilizing invasive synchronized intermittent mandatory ventilation (SIMV) mode, non-invasive nasal intermittent positive pressure ventilation (NIPPV) mode, or withholding support when deemed unnecessary. Sedation was administered as required, primarily using phenobarbital (10–20 mg/kg) or midazolam (1–3 µg/kg/min), and was withheld if not indicated.

### Statistical analysis

Data analysis was conducted using SPSS 27.0 statistical software. Normally distributed measurement data were presented as mean ± standard deviation (SD), and inter-group comparisons were performed using the independent sample *t*-test. Non-normally distributed measurement data were represented as median (Q1, Q3), with inter-group comparisons conducted using the Mann–Whitney *U* test. Categorical data were expressed as cases (%), and analyzed using the *χ*^2^ test for inter-group comparisons. Similarly, ordinal data were reported as cases (%) and compared between groups using the Mann–Whitney *U* test. A two-sided *P*-value of <0.05 was deemed statistically significant.

## Results

### Comparison of baseline clinical characteristics

A total of 72 neonates with perinatal asphyxia were included for transport. After excluding 2 cases in which families abandoned treatment within 24 h of admission, 3 cases where hypothermia treatment was not completed within 72 h, and 2 cases where the age at hypothermia initiation exceeded 24 h, 65 cases were ultimately included (42 males and 23 females), with 17 in the active group and 48 in the control group. Except for the lower 1 min Apgar score in the active group (*P* < 0.05), no statistically significant differences were observed in gestational age, sex, birth weight, mode of delivery, Apgar scores at 5 and 10 min, initial blood gas pH and BE, or HIE severity between the two groups (all *P* > 0.05) (Table 1).

**Table 1:** Baseline characteristics.

Characteristics	Active (*n* = 17), *n* (%)	Control (*n* = 48), *n* (%)	*P*-value
Neonata characteristics			
Gestational age (week)^a^	38.76 ± 1.39	38.96 ± 1.39	0.85
Birth weight (g)^a^	3107.06 ± 563.23	3149.17 ± 537.94	0.964
Male sex	11 (64.7)	31 (64.6)	0.993
Delivery mode			
Vaginal	9 (52.9)	23 (47.9)	0.722
Cesarean	8 (47.1)	25 (52.1)	
Apgar scores^a^			
1 min	2.71 ± 1.16	3.25 ± 1.90	0.009
5 min	4.82 ± 2.09	5.31 ± 2.30	0.582
10 min	6.06 ± 2.04	6.69 ± 2.05	0.659
First blood gas values			
PH^a^	7.05 ± 0.17	7.04 ± 0.20	0.338
Base deficit (mmol/L)^a^	−17.13 ± 6.55	−16.91 ± 6.83	0.746
HIE stage			
Mild	4 (23.5)	9 (18.8)	0.594
Moderate	9 (52.9)	25 (52.1)	
Severe	4 (23.5)	14 (29.2)	

^a^ Data presented as mean ± SD.

### Comparison of transport variables at different stages

**Figure 1:**
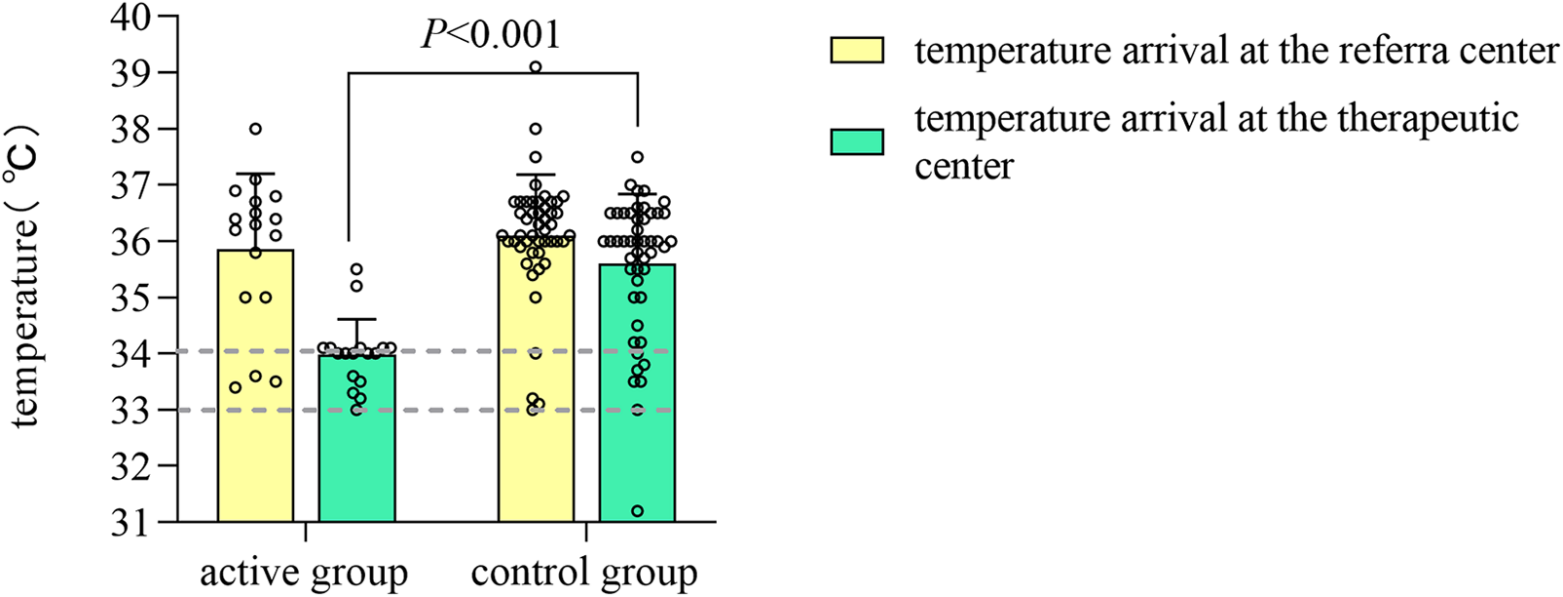
Bar chart of temperature measurements in the active and control groups before and after transport.

In comparison to the control group, the active group exhibited a lower average temperature upon arrival at the treatment center (34.03 ± 0.64°C vs. 35.60 ± 1.23°C, Z = −4.9921, *P* < 0.05). A greater proportion of the active group arrived within the target temperature range [88.2% (15/17) vs. 16.7% (8/48), *χ*^2^ = −0.774, *P* < 0.001] (Figure 1). The median time required to reach the target temperature from the moment of departure from the referral center was reduced by 1.67 h in the active group (*P* < 0.05). Additionally,the temperature difference observed before and after transport was greater in the active group (*P* < 0.05), with no cases of hypothermia (<33°C) occurring in this group; however, one case (2.1%) of hypothermia was reported in the control group. The median age at which both groups initiated hypothermia treatment was within 6 h post-birth, with the active group starting earlier, although these differences were not statistically significant (*P* > 0.05).

The pH and BE values before and after transport were comparable between the two groups, however, changes in BE values were significant (*P* < 0.05), with negative values decreasing, indicating that cooling during transport does not exacerbate acidosis (Table 2).

**Table 2:** Transport characteristics.

Transport variables	Active (*n* = 17), *n* (%)	Control (*n* = 48), *n* (%)	*P*-value
Time profiles			
Stabilization time (min)^b^^,^^e^	35 (30–48)	30 (25–45)	0.233
Age when hypothermia was initiated (h)^b^	3.5 (1–7.5)	4.3 (2–9.8)	0.432
Age when target temperature was achieved (h)^b^	6.5 (3.33–11)	6.925 (4.615–11.925)	0.455
Age when arrived at the therapeutic centre (h)^b^	8 (5–12.6)	4.5 (3–9.875)	0.039
Duration of transport (h)^b^	2.5 (2–6.25)	2 (1.5–3)	0.089
Distance of transport (km)^b^	98 (50.5–399.5)	63 (30–99.25)	0.037
Time from the referral center to the target temperature (h)^b^	1 (0.445–1.5)	2.67 (2.04–4.105)	<0.001
Temperature profiles			
Temperature difference before and after transport (℃)^b^	1.9 (1.25–2.75)	0.3 (−0.1–1.25)	<0.001
Temperature after transport (℃)^a^	34.03 ± 0.64	35.60 ± 1.23	<0.001
Temperature range before transport			0.439
<33℃	0 (0)	0 (0)	0.105
33℃–34℃	3 (17.6)	5 (10.4)	
>34℃	14 (82.4)	43 (88.6)	
Temperature range after transport			
<33℃	0 (0)	1 (2.1)	<0.001
33℃–34℃	15 (88.2)	8 (16.7)	
>34℃	2 (11.8)	39 (81.3)	
Blood gas values			
PH^a^^,^^c^	7.17 ± 0.16	7.15 ± 0.20	0.177
PH^a^^,^^d^	7.34 ± 0.08	7.20 ± 0.57	0.313
Base deficit (mmol/L)^a^^,^^c^	−12.1 ± 6.21	−13.1 ± 6.7	0.823
Base deficit(mmol/L)^a^^,^^d^	−6.83 ± 5.13*	−7.49 ± 5.44*	0.925
Vital signs			
Heart rate (/min)^a^^,^^c^	135.47 ± 24.84	132.67 ± 22.73	0.999
Heart rate (/min)^a^^,^^d^	107.41 ± 14.04	126.02 ± 20.24	0.095
Mean pressure (mmHg)^a^^,^^c^	44.41 ± 6.95	49.17 ± 7.53	0.484
Mean pressure (mmHg)^a^^,^^d^	47.65 ± 8.78	46.44 ± 7.08	0.273

^a^ Data presented as mean ± SD.

^b^ Data presented as median(IQR).

^c^ Before transport at referring centre.

^d^ After transport at the therapeutic centre.

^e^ Time interval from arrival to departure from the referral center.

* Compared with base deficit before transport, *P* < 0.05.

### Comparison of hospitalization outcomes

The median length of stay in the control group was increased by 4 days compared to the active group (*P* < 0.05), and the median duration of mechanical ventilation was extended by 63.5 h in the control group (*P* < 0.05) (Table 3).

**Table 3:** Therapeutic outcomes.

Neonatal outcomes	Active (*n* = 17), *n* (%)	Control (*n* = 48), *n* (%)	*P*-value
Inotropic support	12 (70.6)	37 (77.1)	0.836
Max dopamine(ug/kg/min)^a^	5 (0–10)	5 (3–8)	0.635
Max dobutamine(ug/kg/min)^a^	0 (0–10)	5 (0–5)	0.395
Inhaled nitric oxide use	2 (11.8)	3 (6.3)	0.839
Mechanical ventilation	16 (94.1)	40 (83.3)	0.485
Length of hospitalization (day)^a^	15 (12.5–18.5)	19 (16–26)	0.005
Electrographic seizures	6 (35.3)	17 (35.4)	0.993
Length of ventilation (h)^a^	73 (38.75–131.25)	136.5 (81–199.25)	0.044
Need for gavage feeds at discharge	2 (11.8)	13 (27.1)	0.34
Survival to discharge	15 (88.2)	38 (79.2)	0.642
Mortality^b^	1 (5.9)	6 (12.5)	0.763
MRI^c^			
Complete MRI	17 (100)	41 (85.4)	0.226
Abnormal MRI	12 (70.6)	20 (48.8)	0.128
Areas of brain injury on MRI			
White matter or cortex	11 (64.7)	16 (39)	0.074
Deep grey matter	7 (41.2)	11 (26.8)	0.282
Cerebellum	1 (5)	2 (4)	1

^a^ Data presented as median(IQR).

^b^ Mortality was defined as death prior to hospital discharge.

^c^ MRI, magnetic resonance imaging.

Statistical differences in aEEG grading on day 3 of cooling were observed between the two groups (*P* < 0.05), with a higher proportion of patients in the severe range in the control group [45.8% (22/48) vs. 11.8% (2/17)]. Furthermore,the proportion of neonates in the active group classified in the severe range on day 3 was significantly reduced compared to day 1 (*P* < 0.05), while no statistically significant difference was found in the control group between day 1 and day 3 (*P* > 0.05) (Table 4).

**Table 4:** aEEG grade.

aEEG grade	Active (*n* = 17), *n* (%)	Control (*n* = 48), *n* (%)	*P*-value
First aEEG^a^			
Normal	6 (35.3)	12 (25)	0.627
Morderate	3 (17.6)	12 (25)	
Severe	8 (47.1)	24 (50)	
Third aEEG			
Normal	11 (64.7)	21 (43.8)	0.041
Morderate	4 (23.5)	5 (10.4)	
Severe	2 (11.8)*^,^**	22 (45.8)	
*P*-value	0.009	0.107	

^a^ aEEG, amplitude integrated electroencephalogram.

* Compared with the control group, *P* < 0.05.

** Compared with Day 1 aEEG, *P* < 0.05.

Other assessments of neonatal outcomes, including the use of vasoactive drugs, seizures, distribution of HIE-related cranial MRI injury areas, and mortality rates, revealed no statistically significant differences between the two groups (*P* > 0.05) (Table 3).

## Discussion

Evidence indicates that the duration of the latent period between primary and secondary energy failure is inversely proportional to the severity of primary asphyxia injury. Clinical trials demonstrate that initiating cooling within six hours after birth can significantly reduce the risk of death or disability, establishing HIE as a time-dependent emergency (15–18). International guidelines emphasize that initiating hypothermia within 6 h of birth can maximize its neuroprotective effects (19). Clinical trials have demonstrated that commencing cooling within this time frame can significantly reduce the risk of death or disability (20–23). Furthermore, the earlier cooling is initiated, the more favorable the outcomes tend to be (16, 18, 24). Numerous studies have been conducted internationally that compare cooling protocols for HIE during transport (14, 21, 25–29). Servo-controlled cooling devices are increasingly recommended for neonatal transport owing to their convenience, precision, and stability (7, 21, 23, 26, 28, 30). Our center is the first NICU in the country to utilize active servo-controlled devices for transport, having successfully completed nearly 20 cases of active hypothermic transport for neonates with HIE. However, delays in cooling may occur due to factors such as families’ hesitancy to transfer patients, remote referral centers, and limited medical resources, which result in the inability to promptly assess brain injury and effectively implement therapeutic hypothermia. Many countries, including China, are currently facing the challenge of delayed hypothermia initiation, underscoring the critical need to commence therapeutic hypothermia as early as possible. In this study, however, the active group achieved the target temperature more rapidly. According to Laptook et al., initiating cooling treatment for HIE infants between 6 and 24 h after birth decreases the probability of death or disability by 76% compared to no cooling, with a 64% likelihood of achieving at least a 2% reduction in death or disability at 18–22 months (31). In this study, all neonates initiated therapeutic hypothermia within 24 h. We conducted a detailed analysis comparing cooling methods with and without servo-controlled devices during transport, focusing on the parameters of neonatal transport and the outcomes for the neonates. The results indicate that therapeutic hypothermia during transport is both safe and feasible. Additionally, servo-controlled active cooling facilitates a faster and more stable attainment of the target temperature, resulting in improved EEG outcomes.

Hagan et al. (29) conducted a meta-analysis of eight published studies, demonstrating that the use of servo-controlled cooling devices during transport enables a greater number of infants to reach the target core temperature upon arrival at the treatment center, while also reducing temperature fluctuations during transport (25). Chaudhary reported a success rate of 100% (22), while Akula reported 80% (21), in this study, the rate was 88.2%, which may be related to shorter transport distances in individual cases. Although the median age of the active group upon arrival at the treatment center exceeded 6 h, this may be attributed to longer transport distances. These findings strongly support the feasibility of servo-controlled cooling devices and highlight the necessity for early implementation of hypothermia during transport. This study found that neonates in the active group reached the target temperature more quickly than those in the control group. Despite longer transport distances, a higher proportion of neonates in the active group were within the target temperature range upon arrival, with no incidents of hypothermia, consistent with the results from Nitin Goel et al. (14) The servo-controlled device continuously monitors core temperature via a rectal probe to gradually achieve the set temperature, allowing for precise control. Furthermore, stable core temperatures minimize excessive fluctuations in physiological status, thereby reducing the risk of hypothermia.

If neonates are exposed to a cold environment without being dried, they can lose heat at a rate of 0.2–1°C/min within minutes after birth. Asphyxiated neonates experience a more rapid decline in body temperature compared to non-asphyxiated ones due to reduced heat production. Robertson et al. reported that despite standard rewarming methods, infants in the standard care group could remain in a state of “natural” hypothermia for up to 15 h (32). This study found that the hypothermia rate in the control group was 2.1%, while no incidents occurred in the active group, which was attributed to continuous core temperature monitoring and timely temperature feedback from the servo-controlled device. Hypothermia can lead to complications such as arrhythmias and pulmonary hypertension, which are associated with morbidity and mortality (21, 33). Gloria et al. reported that hypothermia during transport (temperature <33°C) increases the risk of neurological damage before discharge (34). Existing studies have shown that hypothermia increases the demand for inhaled nitric oxide and ECMO treatment, prolongs the duration of oxygen therapy, and is associated with a higher incidence of bradycardia and mortality (35, 36).

In this study, the duration of hospital stay and mechanical ventilation for newborns in the active group were significantly shorter than those in the control group. One possible explanation for this difference is that the initiation of cooling and the achievement of the target temperature occurred earlier in the active group compared to the control group. Although the differences in these two parameters were not statistically significant, they may be influenced by the longer transport distance in the active group and the relatively small sample size. Furthermore, this study is retrospective, and the management of respiratory care and the initiation of enteral nutrition for patients with HIE at our center have gradually evolved, which may account for the observed differences. Additional randomized controlled studies are necessary to confirm the effects of active hypothermia on these outcomes. For instance, a study by Eniko et al. indicated that the median age at which cooling was initiated in the active group was 2.58 h earlier (*P* < 0.0001), and the median age at which the target temperature was reached was 1.83 h earlier (*P* < 0.0001) (27).

Research conducted by Nash (37) and Bourel-Ponchel (38) underscores the critical role of continuous EEG monitoring in patients with HIE. This monitoring technique is effective in accurately detecting electrographic seizures and possesses a high predictive value regarding mortality and neurodevelopmental outcomes in HIE patients. Additionally, Saliba et al. (39) further emphasized the significance of EEG in both the diagnosis and prognostic evaluation of HIE. Thoresen et al. indicated that normalizing aEEG background patterns within 48 h of hypothermia treatment is predictive of improved outcomes for affected infants (40). However, bedside aEEG is not available in most of the district referral hospitals, making the dianosis of HIE incomplete. In fact, we are building integrative aEEG equipments in our transfer platform now, hopefully could move forward the moniter time. The study demonstrates a significant reduction in the proportion of severe aEEG classifications on the third day of hypothermia in the active group compared to the control group, indicating that earlier cooling yields more favorable results. Nicolet Monitor offers convenience for bedside use, unrestricted by time or space, allowing for real-time monitoring of brain activity to assess brain damage and identify seizures. Furthermore, aEEG is particularly user-friendly for clinicians with limited experience. Consequently, it is strongly recommended to conduct continuous EEG monitoring for neonates with HIE during therapeutic hypothermia.

This study does have certain limitations. Firstly, it is a retrospective analysis, which may introduce potential information bias. Secondly, there is an absence of long-term neurodevelopmental follow-up data. Future multi-center, prospective randomized controlled trials are essential to further elucidate the safety and efficacy of active hypothermia during the transport of neonates with HIE.

In conclusion, servo-controlled cooling therapy during transport is both safe and effective. The servo-controlled device provides advantages such as precise temperature regulation and rapid cooling. From the perspective of the transport team, it ensures greater temperature stability with minimal personnel intervention, allowing the team to allocate more time and resources to patient monitoring and care. Medical institutions with suitable facilities and those involved in long-distance transport should consider implementing this method.

## Data Availability

The original contributions presented in the study are included in the article/Supplementary Material, further inquiries can be directed to the corresponding author.
